# CARP-1 Functional Mimetics Are a Novel Class of Small Molecule Inhibitors of Malignant Pleural Mesothelioma Cells

**DOI:** 10.1371/journal.pone.0089146

**Published:** 2014-03-05

**Authors:** Shazia Jamal, Vino T. Cheriyan, Magesh Muthu, Sara Munie, Edi Levi, Abdelkader E. Ashour, Harvey I. Pass, Anil Wali, Mandip Singh, Arun K. Rishi

**Affiliations:** 1 John D. Dingell VA Medical Center, Wayne State University, Detroit, Michigan, United States of America; 2 Karmanos Cancer Institute, Wayne State University, Detroit, Michigan, United States of America; 3 Department of Oncology, Wayne State University, Detroit, Michigan, United States of America; 4 Department of Pathology, Wayne State University, Detroit, Michigan, United States of America; 5 Department of Pharmacology and Toxicology, College of Pharmacy, King Saud University, Riyadh, Kingdom of Saudi Arabia; 6 Division of Cardiothoracic Surgery, New York University Cancer Center, New York, United States of America; 7 College of Pharmacy and Pharmaceutical Sciences, Florida A&M University, Tallahassee, Florida, United States of America; UCSF/VA Medical Center, United States of America

## Abstract

Malignant pleural mesothelioma (MPM) is an asbestos-related thoracic malignancy that is characterized by late metastases, and resistance to therapeutic modalities. The toxic side-effects of MPM therapies often limit their clinical effectiveness, thus necessitating development of new agents to effectively treat and manage this disease in clinic. CARP-1 functional mimetics (CFMs) are a novel class of compounds that inhibit growth of diverse cancer cell types. Here we investigated MPM cell growth suppression by the CFMs and the molecular mechanisms involved. CFM-1, -4, and -5 inhibited MPM cell growth, in vitro, in part by stimulating apoptosis. Apoptosis by CFM-4 involved activation of pro-apoptotic stress-activated protein kinases (SAPKs) p38 and JNK, elevated CARP-1 expression, cleavage of PARP1, and loss of the oncogene c-myc as well as mitotic cyclin B1. Treatments of MPM cells with CFM-4 resulted in depletion of NF-κB signaling inhibitor ABIN1 and Inhibitory κB (IκB)α and β, while increasing expression of pro-apoptotic death receptor (DR) 4 protein. CFM-4 enhanced expression of serine-phosphorylated podoplanin and cleavage of vimetin. CFMs also attenuated biological properties of the MPM cells by blocking their abilities to migrate, form colonies in suspension, and invade through the matrix-coated membranes. Both podoplanin and vimentin regulate processes of cell motility and invasion, and their expression often correlates with metastatic disease, and poor prognosis. The fact that phosphorylation of serines in the cytoplasmic domain of podoplanin interferes with processes of cellular motility, CFM-4-dependent elevated phosphorylated podoplanin and cleavage of vimentin underscore a metastasis inhibitory property of these compounds, and suggest that CFMs and/or their future analogs have potential as anti-MPM agents.

## Introduction

Malignant pleural mesothelioma (MPM) is a lethal asbestos-related malignancy [1]. Scores of workers have been exposed to asbestos throughout world. Since asbestos exposure has been identified as a risk factor in diseases including asbestosis, lung cancer and MPM [1], it is estimated that approximately 2,000–3,000 people will be diagnosed as MPM patients each year in the US. Although the use of asbestos has been significantly curtailed, the incidence of asbestos-related diseases including MPM is expected to continue in the next decade in the United States and Europe [3], [4]. The multimodality treatment for MPM in the clinic often consists of surgery, adjuvant or neoadjuvant chemotherapy, and radiation [2]. Most chemotherapeutic agents are not very effective against MPM, with typical single-agent response rates of ≤20% [5]. The median survival of MPM patients ranges from 9–17 months, and remains unacceptably low [3]. Development of novel treatment strategies for MPM is therefore warranted to improve the survival outcome in patients and overcome resistance to currently available chemotherapies.

CARP-1, also known as CCAR1, is a peri-nuclear phospho-protein that is a regulator of cancer cell growth and apoptosis signaling [6]–[8]. In addition to being a key transcriptional co-activator of p53 in regulating adriamycin (ADR)-dependent DNA damage-induced apoptosis, deprivation of serum growth factors also resulted in elevated CARP-1 expression [6]–[8]. Antisense-mediated depletion of CARP-1, on the other hand, abrogated cancer cell growth inhibition by ADR [6]. The apoptosis signaling by EGFRs stimulated tyrosine phosphorylation of CARP-1 and targeted CARP-1 tyrosine^192^, while CARP-1-dependent apoptosis in turn involved activation of SAPK p38 and caspase-9 [8]. Recent studies further revealed that protein kinase A (PKA) inhibitor H89 attenuates human breast cancer (HBC) cell growth in part by targeting CARP-1 threonine^667^-dependent suppression of c-Myc transcription [9]. Phosphopeptide mapping studies indicate that CARP-1 is also a serine phospho-protein, and the epidermal growth factor (EGF) as well as the ATM kinase signaling phosphorylates specific serine residues of CARP-1 [10]–[12].

The Anaphase Promoting Complex/Cyclosome (APC/C) is a multiprotein complex with E3 ubiquitin ligase activity [13]. Dysregulation of APC/C may be associated with tumorigenesis since many APC/C-targeting/activating molecules such as securin, polo-like kinase, aurora kinase, and SnoN are potential oncogenes [14]. A yeast-two-hybrid (Y2H) screening assay revealed CARP-1 interaction with APC-2 protein. Following mapping of epitopes involved in CARP-1 binding with APC-2, we developed a fluorescence polarization (FP) based in vitro binding assay. High through-put screening of a chemical library in conjunction with this FPA yielded multiple, small molecule inhibitors (SMIs) of CARP-1/APC-2 binding, termed CARP-1 Functional Mimetics (CFMs) [15]. Here we investigated MPM growth inhibition by CFMs. CFMs inhibit MPM cell growth in part by stimulating apoptosis while impacting the motility and invasion signaling and biological properties of colony formation, invasion, and migration of the MPM cells. Our proof-of-concept studies presented here show that pharmacologically-active CFMs are suppressors of MPM cell growth, and suggest that CFMs or their derivatives could provide novel means to combat/treat resistant MPM.

## Methods

### Materials

DMEM, Ham's F-12 medium and fetal bovine serum (FBS) was purchased from Life Technologies, Inc., Grand Island, NY. CFM-1, 4, and 5 were obtained from ChemDiv and/or ChemBridge, San Diego, CA, and dissolved in dimethyl sulfoxide (DMSO) at a stock concentration of 10, 50, and 50 mM, respectively, and stored at −20°C. Appropriate working concentrations were prepared with the cell culture medium immediately before the experiments. Cisplatin, DMSO, chemicals including 3–4, 5-dimethyltiazol-2-yl-2.5-diphenyl-tetrazolium bromide (MTT), cremophor and anti β-Actin mouse monoclonal antibody were obtained from Sigma Aldrich, St. Louis, MO. The monoclonal antibodies for ABIN2, vimentin and c-myc, and the polyclonal antibodies for c-Jun, DR5, and DR4 proteins were obtained from Santa Cruz Biotech, Santa Cruz, CA. The mouse monoclonal antibody for α-tubulin and phospho-serine monoclonal antibody 16B4 were obtained from Calbiochem (Billerica, MA) and Enzo Life Sciences (Farmingdale, NY), respectively. Anti-cyclin B1, anti-caspase-3, anti-phospho-JNK (Threonine183/Tyrosine 185) G9 mouse monoclonal antibodies, anti-JNK (56G8) rabbit monoclonal antibody, and rabbit polyclonal antibodies for PARP, phospho and total p38 SAPK, ABIN1, IκBα, and IκBβ proteins were obtained from Cell Signaling Technology (Beverly, MA). Anti-p21 Rac1 mouse monoclonal antibody was purchased from BD Biosciences, San Jose, CA. Anti-podoplanin D2-40 mouse monoclonal (SIG-3730; antigen M2A) and rat monoclonal [Clone NZ-1.2; antigen: synthetic peptide corresponding to amino acids 38–51 (EGGVAMPGAEDDVV) of podoplanin] were purchased from Covance (Dedham, MA) and Imgenex (San Diego, CA), respectively. Anti-Ubiquitin, Lys63-Specific (Clone Apu3), and Anti-Ubiquitin, Lys48-Specific, (Clone Apu2) rabbit monoclonal antibodies were obtained from Millipore, Temecula, CA. Generation and characterization of the anti-CARP-1/CCAR1 rabbit polyclonal antibodies have been described before [6]. Enhanced Chemi-luminescence Reagent was purchased from Amersham Biosciences (Piscataway, NJ) and the Protein Assay Kit was purchased from Bio-Rad Laboratories (Hercules, CA).

### Cell Lines and Cell Culture

Isolation, establishment and characterization of the human MPM cell lines H2373, H2714, and H2461 has been described before [16]. Murine mesothelioma AB12 cells were kindly provided by Dr. Steven Albelda, University of Pennsylvania Medical Center, Philadelphia, PA, and have been described before [17]. Human MPM cells were routinely cultured in DMEM supplemented with 10% FBS, 100 units/ml of penicillin, and 100 µg/ml of streptomycin. AB12 cells were cultured in high-glucose DMEM supplemented with 10% fetal bovine serum, 100units/mL of penicillin, and 100 µg/mL of streptomycin. Cells were maintained at 37°C and 5% CO_2_. For cell growth and apoptosis studies, the MPM cells were cultured in fresh media with 5% FBS prior to their treatments with various agents.

### Immuno-cytochemical labeling

For immuno-cytochemical analyses approximately 5×10^3^ cells were seeded onto a slide well, and allowed to grow overnight at 37°C incubator. The cells were then either untreated, treated with 5 µg/ml Cisplatin, 20 µM CFM-1, 10 µM each of CFM-4 or CFM-5 for 12 or 24 h. The slides were rinsed to remove the media, and the cells fixed for staining using a 1∶250 dilution of anti-CARP-1 (α2) or anti-c-myc antibodies, or 1∶500 dilution of the anti-phospho-p38, DR5, or D2-40 antibodies. The antibody-stained cells were then photographed under different magnifications utilizing Zeiss microscope with attached 35 mm camera for recording the photomicrographs.

### Western Immuno-blotting, Immunoprecipitation, MTT and apoptosis Assays

Logarithmically growing cells were treated with Cisplatin or CFM compounds, and cells were lysed to prepare protein extracts. For western immuno-blotting (WB) analyses, cells were harvested and lysed in RIPA buffer (50 mM Tris-HCI, pH 8.0, 150 mM sodium chloride, 1.0% NP-40, 0.5% sodium deoxycholate, 0.1% sodium dodecyl sulfate (SDS), and 0.1% of protease inhibitor cocktail) for 20 min at 4°C. The lysates were centrifuged at 14,000 rpm at 4°C for 15 min to remove debris. Protein concentrations of whole cell lysates were determined using the Protein Assay Kit. For immunoprecipitation, ∼1 mg of proteins from untreated or treated cell lysates were first incubated with anti-phospho-serine monoclonal antibody 16B4 that specifically recognizes phosphorylated serine residues that are immediately followed by lysine (pSK, substrate for CDC kinase) or proline (pSP, substrate for MAP/SAP kinases) essentially following methods described before [8]. The protein extracts (50 or 100 µg) or immunoprecipitates were electrophoresed on 9–12% SDS-polyacrylamide gels and transferred to polyvinylidene difluoride (PVDF) membrane (Bio-rad, Hercules, CA) essentially as described before [6], [8]. The membranes were hybridized with primary antibodies followed by incubation with appropriate secondary antibodies. The antibody-bound proteins were visualized by treatment with the chemiluminescence detection reagent (Amersham Biosciences) according to manufacturer's instructions, followed by exposure to X-ray film (Kodak X-Omat). The same membranes were re-probed with the anti-β actin or anti-α-tubulin antibody, which was used as an internal control for protein loading.

The cell growth inhibition was assessed by using MTT assay. Briefly, a stock solution of 5 mg/ml of MTT was prepared in sterile 1× PBS, filtered through 0.2 µm filter, and stored at 2–8°C. 4–5×10^2^ cells were seeded in 96-well plates. After incubation with or without agents, MTT stock solution equal to one tenth of the original culture volume was added to each culture, followed by incubation of cells at 37°C for further 2 h. At the end of the incubation, the media was removed and cells were treated with 100–200 µl of DMSO to solubilize the dye. The assessment of the live cells was derived by measuring the absorbance of the converted dye at wavelengths of 490 to 570 nm.

Apoptosis levels were determined by staining for fragmented DNA utilizing terminal deoxynucleotidyl transferase-mediated nick end labeling (TUNEL) assay kit (Roche Diagnostics, Indianapolis, IN) essentially following manufacturer suggested protocols [6], [8]. The cells were treated with various agents, fixed, labeled and photographed essentially as detailed in immuno-cytochemical staining methods above.

### Cell Migration, Invasion, and Clonogenic Assays

The effects of CFMs on migration of MPM cells were measured by the “scratch” assay. Cells were grown in a 6-well plate (∼10,000 cells/well), and a scratch was created in the cell monolayer using sterile pipette tip. The cells were then allowed to grow in the absence (Control) or presence of 10 µM dose of each of the CFMs for a period of 72–96 h. Images were captured at the beginning and at regular intervals during cell migration to close the scratch, and the images were compared to quantify the migration rate of the cells essentially as described before [18]. The cells were photographed under different magnifications utilizing Zeiss microscope with attached 35 mm camera for recording the photomicrographs.

Clonogenic assay: Cells were sandwiched between 0.6% and 0.3% agarose in DMEM medium containing 5% FBS in a six-well chamber (500 cells/chamber), and treated with buffer (Control), or respective CFM (10 µM) for 9 days at 37°C humidified CO_2_ incubator. The colonies from multiple random fields were counted, compared to control and photographed essentially as above.

Invasion assay: Basement membrane is a thin extracellular matrix (ECM) that underlies epithelia and endothelia and separates epithelial cancer cells from the stroma. Tumor cells produce proteases that degrade ECM to cross the basement membrane to invade stroma and establish distant metastases. The in vitro Boyden Chamber assay (Chemicon International, CA) using Matrigel is the most reliable, reproducible, and representative of in vivo invasion. In this assay, cancer cells are placed in the upper chamber that is separated from the lower chamber by a porous membrane coated with Matrigel [18], [19]. Briefly, pre-warmed serum free medium (300 µl) was used to hydrate the ECM layer of each chamber for 15–30 minutes at room temperature. Approximately 2–2.5×10^5^ MPM cells were seeded in the upper chamber in a serum-free medium without or with 10 µM dose of the respective CFM. The lower chamber was supplied with medium containing 10% FBS that served as chemo-attractant to stimulate migration. After an interval, tumor cells present on the lower side of the membrane in the lower chamber were stained, and photographed as above. In addition, the stained cells from the lower side of membrane of some wells were dissociated, lysed in a buffer, followed by quantitation using a fluorescence plate reader with 480/520 nm filter set. The measurements were then plotted as bars in histogram.

### Detection of MMP expression in human MPM cells

H2373 MPM cells were separately treated with CFM-4 or CFM-5, followed by their homogenization in RIPA buffer (500 µl of lysis buffer per 1×10^6^ cells). The cell lysates were centrifuged at 10,000× g for 5 min, and the protein concentration in the supernatant of the respective lysate was determined by using Bicinchoninic acid assay. The lysates were stored at −80°C until further use. MMP activation in each lysate was measured using the Quantibody reverse phase human MMP array kit according to manufacturer's instructions (RayBiotech, Norcross, GA). Fluorescence images were detected using a GenePix 4100A Scanner, and data was analyzed using the QAH-MMP-1 GAL software based on the instruction provided by the array manufacturer.

### Statistical analysis

Where appropriate, statistical analysis was performed using unpaired Student's t-test. A p value less than 0.05 between treatment groups was considered significantly different.

## Results

### CFMs inhibit MPM cell proliferation in part by stimulating apoptosis

**Figure 1:**
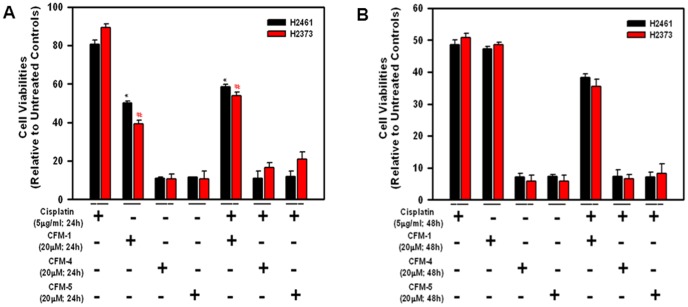
CFMs reduce viabilities of the human MPM cells. Cells were treated with vehicle (Untreated Control), indicated doses of Cisplatin, various CFMs, or a combination of Cisplatin and CFMs for 24 h (A) or 48 h (B). Determination of viable/live cells was carried out by MTT assay. The data in the histograms represent means of three independent experiments; bars, S.E. * and #, p = <0.05 relative to Untreated Control (A). Note that the Y-axis scale is different in panel B.

**Figure 2:**
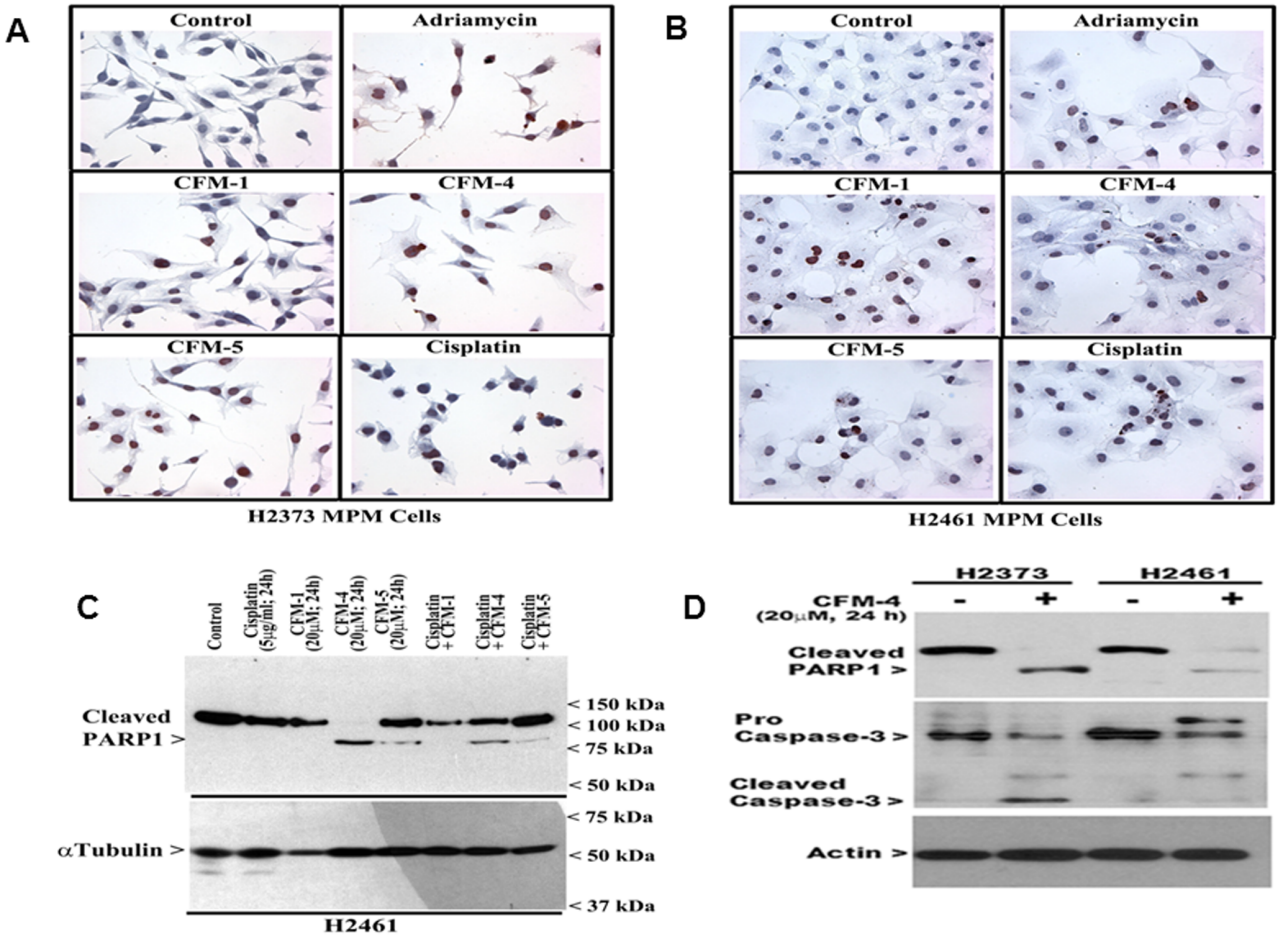
CFMs stimulate apoptosis in MPM cells. (A, B) Indicated MPM cells were either untreated (Control), treated with 2.5 µg/ml Adriamycin, 5 µg/ml Cisplatin or 20 µM dose of respective CFMs for 24 h. Staining of the cells was performed using TUNEL assay as detailed in Methods. Dark brown staining represents fragmented cell nuclei. (C, D) MPM cells were either untreated (denoted as Control in panel C and – in panel D) or treated (denoted as+in panel D) with indicated agents for noted time and dose, and levels of cleaved PARP, pro- and cleaved (activated) caspase-3, and actin proteins were determined by Western blotting.

We have previously found that viabilities of a number of MPM cells were affected following their treatments with CFM-1, -4, or -5 [15]. A majority (∼60–70%) of MPM are characterized as epithelioid histotype. Additional histotypes include sarcomatoid (10–15%) and biphasic/mixed (10–15%) MPM, and are generally more aggressive tumors with poor outcomes. Since Cisplatin is often used as a frontline therapy for MPM in clinic, here we utilized H2461 (epithelioid histologic origin) and H2373 (sarcomatoid histologic origin) MPM cells [16] in a proof-of-concept study to further investigate anti-MPM efficacies of CFMs, and to determine whether CFMs are superior to Cisplatin in inhibiting MPM cell growth. As expected, CFM-1, 4, 5, or Cisplatin inhibited the viability of both the cell lines in a time-dependent manner (Figure 1). In general, the cells were more sensitive to inhibition by all the CFMs when compared with Cisplatin. A 20 µM dose of CFM-1 for 24 h period resulted in approximately 50% growth inhibition of both the MPM cells (Figure 1A). A 20 µM dose of CFM-4 or CFM-5 over a 24 h treatment elicited ∼90% loss of viabilities of both the MPM cells (Figure 1A). Cisplatin treatments (5 µg/ml) over 24 h and 48 h periods however resulted in a modest 10–20% and 50% loss of MPM cell viability, respectively (Figure 1A, B). MPM cells treated with a combination of Cisplatin and either of the CFMs failed to elicit a higher level of growth inhibition. We next investigated whether CFMs promoted apoptosis to inhibit MPM cell growth. Given that a 24 h treatment of both the MPM cells with 20 µM dose of respective CFM resulted in a significant, 50–90-% loss of their viabilities, we chose to utilize a 20 µM dose of each compound to determine induction of apoptosis and the underlying molecular mechanism(s). For immunocytochemical analyses, the MPM cells were directly cultured in 8-well chamber slides and were either untreated or treated with respective CFMs, chemotherapeutic agents Adriamycin (ADR; 2.5 µg/ml) and Cisplatin (5 µg/ml) for a period of 24 h as detailed in Methods. Treatment of the MPM cells with each of the agents resulted in elevated number of TUNEL-positive cells (Figure 2A, B). Additional WB analysis revealed elevated cleavage of caspase-target poly(ADP-ribose) polymerase (PARP) following 24 h treatment of H2461 MPM cells. Although exposure to Cisplatin or CFMs caused reduced levels of PARP1 protein, treatments with the 20 µM dose of CFM-4 or CFM-5 however resulted in a robust cleavage of PARP-1 in the MPM cells (Figure 2C, D). We have previously found that activation of caspases was necessary for transduction of CFM-4-dependent growth inhibitory signaling in HBC cells [15]. Since CFMs promoted PARP cleavage in MPM cells (Figure 2C), we next determined whether caspases were activated following exposure of the MPM cells to CFMs. As shown in figure 2D, treatment of the MPM cells with CFM-4 resulted in elevated levels of cleaved (activated) caspase-3. The data in figures 1 and 2 therefore suggest that CFMs are novel and superior inhibitors of MPM cell growth when compared with conventional anti-MPM therapeutic Cisplatin, and all the three CFMs attenuate MPM cell growth in part by stimulating apoptosis.

### Apoptosis signaling by CFMs involves activation of pro-apoptotic stress-activated protein kinases (SAPKs), and elevated expression of a novel transducer of apoptosis CARP-1/CCAR1

**Figure 3:**
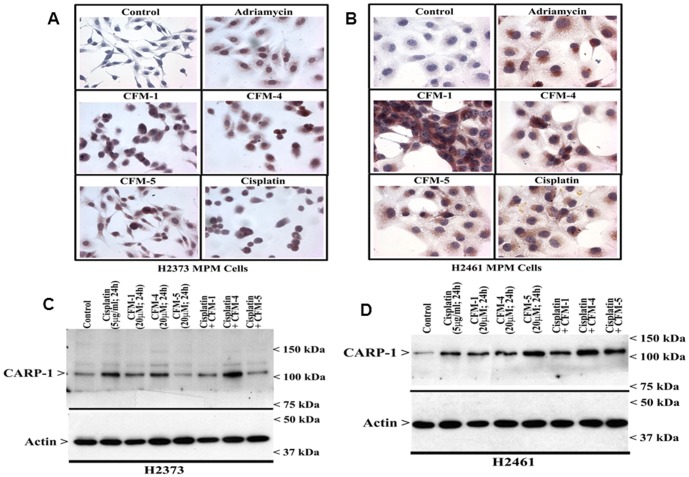
CFMs enhance expression of pro-apoptotic CARP-1. (A, B) Indicated MPM cells were either untreated (Control), treated with Adriamycin, Cisplatin, or respective CFMs as in figure 2. Staining of the cells was performed using anti-CARP-1 (α2) antibody as detailed in Methods. Presence of CARP-1 is indicated by intense brown staining in the nuclei and cytosol of the treated cells. (C, D) MPM cells were either untreated (Control) or treated with different agents for indicated dose and time, and cell lysates were analyzed by western blotting for levels of CARP-1 and actin proteins as in Methods.

CARP-1/CCAR1 has been previously reported by our laboratory and others as a novel and biphasic transducer of cell growth and apoptosis signaling. While CARP-1 functions as a co-activator of nuclear, steroid/thyroid transcription factors, it also functions as a key regulator of p53 function and a transducer of apoptosis signaling by DNA damaging agents such as ADR [6], [7]. Since both the MPM cells displayed increased sensitivity to inhibition by CFM-4 and CFM-5, we next determined whether treatments of MPM cells to these compounds altered CARP-1 expression. In the first instance, the H2373 and H2461 cells were either untreated or treated with ADR, Cisplatin, CFM-1, CFM-4, or CFM-5 as in figure 2, and CARP-1 levels were analyzed by immuno-cytochemical staining utilizing anti-CARP-1 α2 antibodies as noted in Methods. Exposure to CFMs or the chemotherapeutic agents ADR and Cisplatin resulted in increased staining for CARP-1 in both the MPM cells (Figure 3A, B). Consistent with elevated CARP-1 levels following treatments with these agents, WB analysis of the lysates derived from Cisplatin, CFM-1, CFM-4, or CFM-5-treated MPM cells revealed a robust increase in CARP-1 expression when compared with its levels in the lysates from the untreated control cells (Figure 3C, D). However, increase in CARP-1 levels in the cells treated with Cisplatin in combination with either of the CFMs was not significantly different than that noted in the case of the cells treated with each agent alone. Together with data in figure 1 where Cisplatin in combination with CFMs failed to cause increased growth inhibition of MPM cells when compared with either agent alone, the data suggest for a likely overlap of the molecular mechanisms of MPM growth suppression by Cisplatin and CFMs.

**Figure 4:**
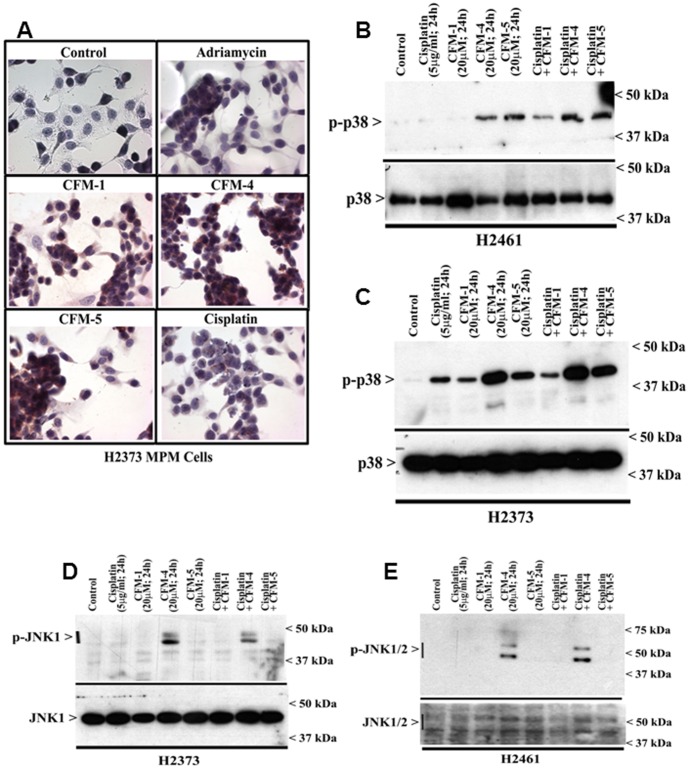
CFMs activate pro-apoptotic SAPKs in MPM cells. (A, B) Indicated MPM cells were either untreated (Control), treated with Adriamycin, Cisplatin, or respective CFMs as in figure 2A. Staining of the cells was performed using anti-phospho-p38 antibody as detailed in Methods. Presence of p38 is indicated by intense brown staining in the nuclei and cytosol of the treated cells. MPM cells were either untreated (Control) or treated with indicated agents for noted time and dose, and levels of phosphorylated p38 (noted as p-p38), and total p38 proteins (B, C) or phosphorylated JNK (noted as p-JNK1/2), and total JNK proteins (D, E) were determined by Western blotting essentially as in figure 2.

Our previous studies have noted a requirement for p38 SAPK in CARP-1-dependent apoptosis signaling [8]. Moreover, our recent reports have revealed that CFM-4 that binds with CARP-1, promoted apoptosis in part by increasing CARP-1 levels and p38 activation in a number of cancer cells including the HBC and medulloblastomas [15], [20]. Here we tested the extent MPM cell inhibitory signaling by CFMs involved activation of SAPKs. We first determined whether and to what extent CFMs can stimulate p38 activation in the MPM cells. Immuno-cytochemical and WB analyses were performed to determine CFM-mediated changes in p38 activation. Treatments with CFM-1, -4, or -5 resulted in elevated staining for phosphorylated (activated) p38 in H2373 cells (Figure 4A) when compared with their untreated counterparts. WB analysis of the cell lysates corroborated a robust activation of p38 in CFM-4, or CFM-5-treated H2461 (Figure 4B) and H2373 (Figure 4C) MPM cells when compared with their untreated controls. Further, WB analysis of the cell lysates revealed activation of JNK1/2 SAPK in the CFM-4-treated MPM cells while treatments of MPM cells with Cisplatin, CFM-1 or CFM-5 did not activate JNK1/2 (Figure 4D, E). These data indicate that although all the CFMs inhibit MPM cell growth in part by stimulating apoptosis, the molecular mechanisms of apoptosis signaling by CFM-4 are likely distinct from that of the CFM-1, CFM-5 or Cisplatin.

**Figure 5:**
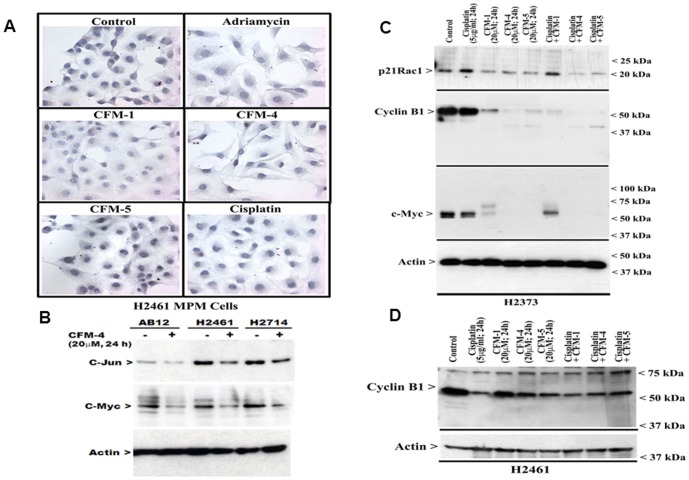
CFMs suppress expression of transducers of MPM cell growth and survival. (A–C) CFM treatments result in loss of c-myc in MPM cells. In panel A, cells were either untreated (Control), treated with Adriamycin, Cisplatin, or respective CFMs as in figure 2A, followed by staining of cells using anti-c-myc antibody as detailed in Methods. Presence of c-myc is indicated by intense brown staining in the nuclei of the untreated cells. (B–D) MPM cells were either untreated (denoted as Control in panels C and D, and – in panel B) or treated (denoted as+in panel B) with indicated agents for noted time and dose, and cell lysates were analyzed by western blotting for levels of c-Jun (panel B), c-myc (panels Band C), and cyclin B1 (panels C and D) and actin proteins as in Methods.

Our earlier studies have also indicated that in addition to stimulating CARP-1 levels and SAPK activation, CFM-4 treatments induced loss of a number of key cell cycle and cell growth regulatory molecules. In particular, levels of mitotic cyclin B1, cell growth and motility regulatory small GTP-binding protein p21Rac1, and oncogene c-myc were diminished in CFM-4-treated cells [15]. In light of these observations, we further investigated whether treatments of MPM cells with CFMs also results in loss of cyclin B1, p21Rac1, and c-myc proteins. As shown in figure 5A, reduced immuno-cytochemical staining for c-myc was noted in H2461 MPM cells that were treated with ADR or CFMs. Consistent with these findings, reduced levels of oncogenes c-jun and c-myc were noted in the lysates of CFM-4-treated human and murine MPM cells by WB analysis (Figure 5B). Additional WB analysis further supported down-regulation of c-myc in MPM cells that were treated with CFMs, but not Cisplatin (Figure 5C). CFM-1, CFM-4 or CFM-5 treatments also suppressed expression of cyclin B1 and p21Rac1 proteins in MPM cells (Figure 5C, D).

### CFMs activate NF-κB signaling in MPM cells

**Figure 6:**
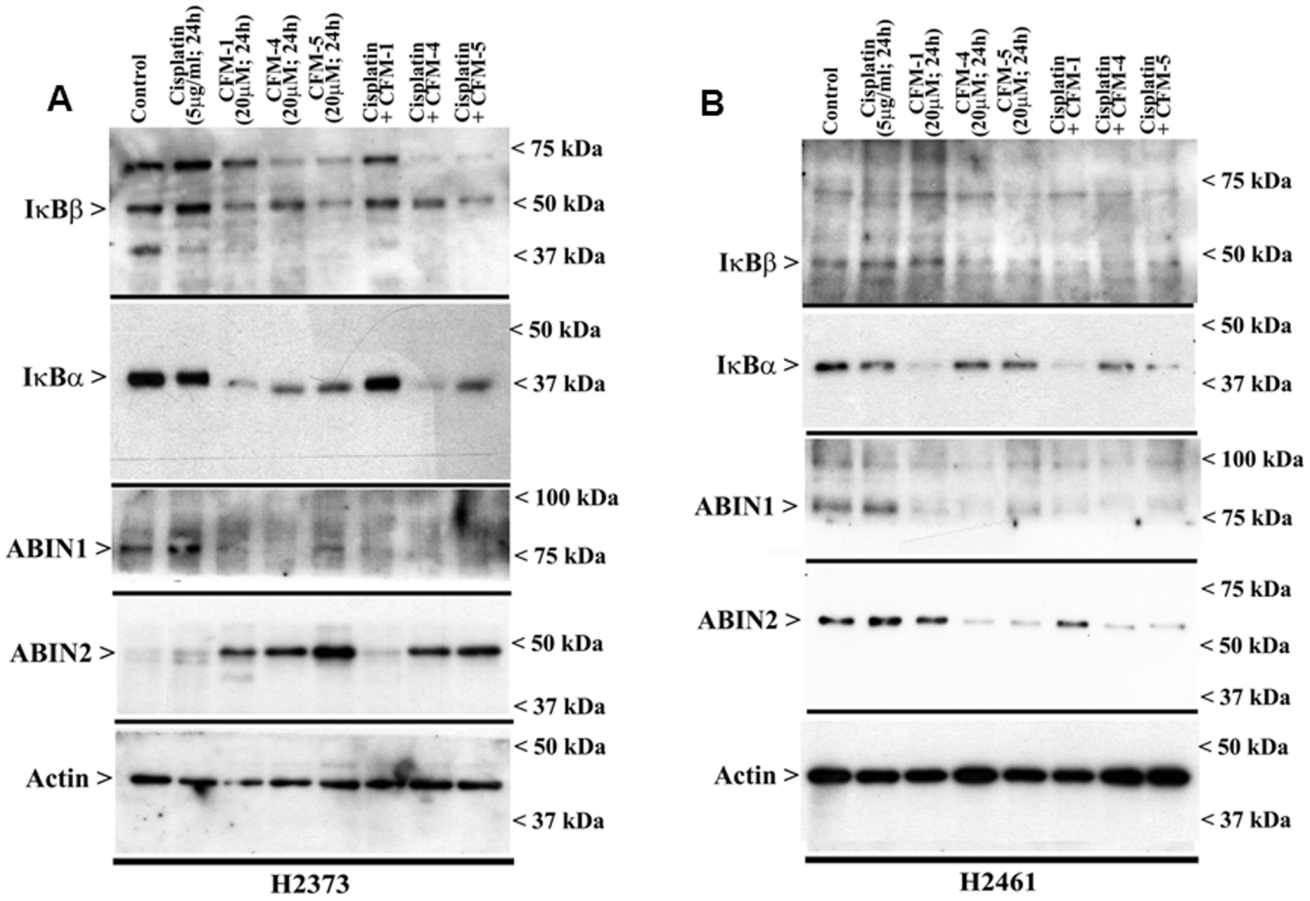
CFM-4 but not Cisplatin activates canonical NF-κB signaling. H2373 (A) and H2461 (B) MPM cells were either untreated (Control) or treated with noted agents for indicated dose and time, and cell lysates were analyzed by western blotting for levels of ABIN1, ABIN2, IκBα, IκBβ, and actin proteins as indicated in Methods.

The NF-κB family of proteins and their signaling is well known to play a crucial role in organismal physiology and pathologies such as chronic inflammation and cancer. Activation of pro-apoptotic MAPKs (p38 or JNK) on the other hand serves to attenuate NF-κB activation in different stress-induced apoptotic contexts [21], [22]. A number of recent studies have revealed a pro-apoptotic role for NF-κB signaling [23]–[25], and together with our recent observations indicating that prolonged exposure of medulloblastoma cells to CFM-4 also resulted in NF-κB activation that likely serves to potentiate/support apoptosis [20]; we investigated the extent CFMs also regulated NF-κB signaling in MPM cells. Our data in figure 6 show that treatments of MPM cells with CFM-1, CFM-4, or CFM-5 results in a diminished levels of NF-κB inhibitory IκBα and/or IκBβ proteins suggesting that these compounds likely activate NF-κB in MPM cells. However, Cisplatin treatments of MPM cells failed to diminish expression of NF-κB inhibitory IκBα and/or IκBβ proteins (Figure 6A, B).

A range of extrinsic and intrinsic signals regulate cell growth and survival by modulating canonical NF-κB signaling [26]. In the canonical pathway, levels of NF-κB inhibitory IκBα and/or IκBβ proteins are regulated by upstream kinase IKK that itself is subject to control by inhibitory molecules such as ABIN1 and 2 proteins [27], [28]. Although ABIN2 levels were significantly up-regulated in CFM-treated H2373 cells, CFM-4 or CFM-5 treatments promoted loss of ABIN2 expression in H2461 cells (Figure 6A, B). ABIN1 expression however was consistently down-regulated in both the MPM cells following their treatments with each of the CFMs (Figure 6). It is also of note here that Cisplatin treatments failed to alter levels of either of the ABIN1, or ABIN2 proteins.

**Figure 7:**
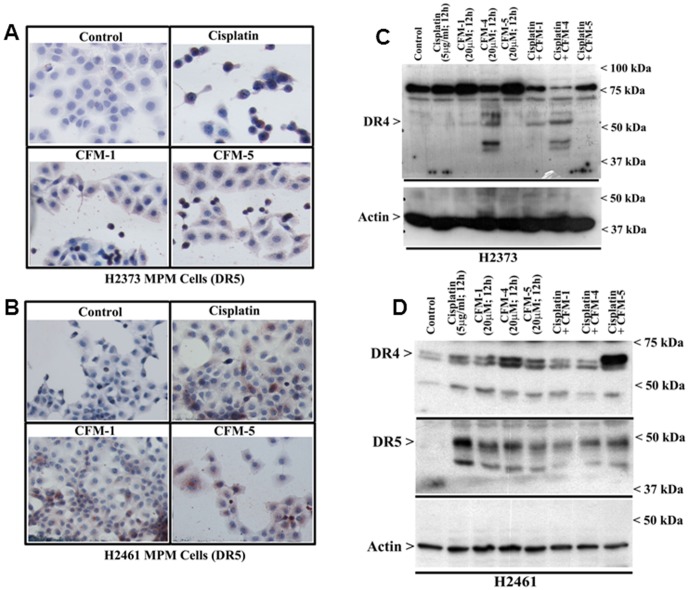
CFMs stimulate expression of cell death signaling death receptors (DRs) 4 and 5 in MPM cells. (A, B) Indicated MPM cells were either untreated (Control), treated with Cisplatin, or respective CFMs as in figure 2A. Staining of the cells was performed using anti-DR5 antibody as detailed in Methods. Presence of increased DR5 is indicated by intense brown staining in the cytosol of the treated cells. MPM cells were either untreated (Control) or treated with indicated agents for noted time and dose, and levels of DR4, DR5 and actin proteins (C, D) were determined by Western blotting essentially as in figure 2.

The tumor necrosis factor (TNF) family of cytokines that include TNF-related apoptosis-inducing ligand (Apo2L/TRAIL) regulate apoptosis by binding to five TNF receptor superfamily members [29]. TRAIL binding to the death receptor 4 (DR4, TRAIL-R1, TR1) and death receptor 5 (DR5, TRAIL-R2, TR2) activates apoptosis signals through the cytoplasmic death domains of the DR4 and 5 receptors. Three additional TRAIL receptors, decoy receptor 1 (DcR1), decoy receptor 2 (DcR2), and soluble osteoprotegerin (OPG), lack the ability to initiate apoptosis and function as inhibitory receptors [30]–[33]. Activation of apoptosis signaling by DRs remains an attractive strategy for therapeutic applications since recombinant human (rh)Apo2L/TRAIL and agonistic antibodies directed against either DR4 or DR5 have shown activity in vitro and in vivo against a range of cancers, and combinations of DR agonists with conventional chemotherapeutics have shown promise in preclinical testing [34]–[37]. Although a variety of normal and cancer cells express DR4 and DR5, and TRAIL has been shown to activate apoptosis signaling in cancer cells, it is likely that elevated levels of DR4 and/or DR5 could sensitize cancer cells to apoptosis by chemotherapy alone or in combination with DR agonists. Since caspase-8 activation is an early and key requirement for apoptosis following DR activation [38], and the fact that caspase-8 activation was also required for apoptosis by CFM-4 in HBC cells [15], we investigated whether MPM cell growth suppression by CFMs involved DRs. Immunocytochemical analysis revealed increased staining for the DR5 protein in MPM cells that were treated with Cisplatin, CFM-1 or CFM-5 (figure 7A, B). The western immunoblot analysis (figure 7C, D) further confirmed elevated expression of DR4 and DR5 proteins in Cisplatin or CFM-treated MPM cells. To the extent, elevated levels of DR4 and DR5 contributed to anti-MPM effects of CFMs remain to be clarified. However, activation of pro-apoptotic p38, and elevated levels of CARP-1, DR4, and DR5 proteins indicates for an overlap of the MPM growth inhibitory molecular mechanisms utilized by Cisplatin and CFMs. The fact that CFM-4 also activates pro-apoptotic JNK1/2 and NF-κB (Figures 4, 6), and suppress levels of p21Rac1, c-myc and cyclin B1 proteins (figure 5), would suggest that CFM-4 utilizes additional MPM inhibitory molecular mechanisms that are distinct from those activated by Cisplatin that could be responsible for its superior anti-MPM efficacies noted in figure 1.

### CFM-4 inhibits MPM cell motility, migration, colony formation, and invasion

We next investigated whether CFMs inhibit biological properties of migration, invasion and colony formation by the MPM cells, and the molecular mechanisms involved. A variety of cell surface and intracellular proteins in conjunction with factors in the extracellular matrix regulate motility and invasive properties of the cancer cells. A number of studies have revealed that a sialomucin type I transmembrane glycoprotein, podoplanin, regulates processes of cell migration, epithelial-to-mesenchymal transition (EMT), and tumor metastasis, as well as cell cycle and cell proliferation through extracellular matrix signaling, and is often related to tumor malignancy and poor outcome in many cancers including MPM [39]–[44]. In addition to podoplanin, a type III intermediate filament cytoskeletal protein, vimentin, that regulates cytoskeletal interactions such as adhesion, migration and signaling, is also frequently overexpressed in invasive cancer cells and associates with metastasis and poor prognosis [45]–[48]. In light of the recent reports that have highlighted anti-MPM intervention strategies that target podoplanin or vimentin [49]–[51], we next determined whether CFMs modulated expression of podoplanin and vimentin proteins in the MPM cells.

**Figure 8:**
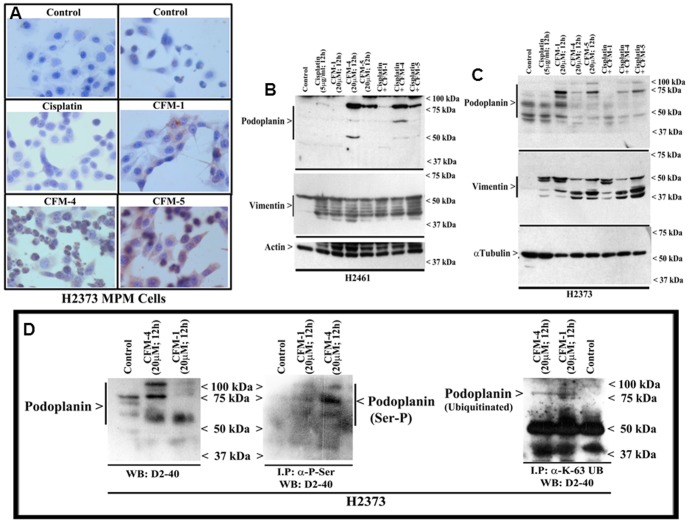
CFM-4 elevates expression and serine phosphorylation of podoplanin. (A) Indicated MPM cells were either untreated (Control), treated with Cisplatin, or respective CFMs as in figure 2A. Staining of the cells was performed using anti-podoplanin D2-40 antibody as detailed in Methods. Presence of increased podoplanin is indicated by intense brown staining in the cytosol of the CFM-1, CFM-5, and CFM-4-treated cells. MPM cells were either untreated (Control) or treated with indicated agents for noted time and dose, and levels of podoplanin, vimentin, actin and α-tubulin proteins (B, C) were determined by Western blotting essentially as in figure 2. (D) H2373 cells were either untreated (Control) or treated with agents as indicated. The cell lysates (1 mg of protein) were first subjected to immunoprecipitation using anti-phospho-serine, anti-Ubiquitin (lys48-specific), or anti-Ubiquitin (lys63-specific) antibodies as in methods. The membranes with cell lysates (50 µg/lane; blot on the left) or the immunoprecipitates (blot on the right) were probed with anti-podoplanin D2-40 antibody as in panel C.

Immunocytochemical analysis of MPM cells treated with CFM-1, CFM-4, or CFM-5 in conjunction with D2-40 anti-podoplanin antibody revealed higher levels of podoplanin when compared with their untreated or Cisplatin-treated counterparts (figure 8A). Consistent with the immunocytochemical analysis, western blot data failed to show elevated levels of podoplanin peptides in Cisplatin-treated cells (Figure 8B, C). MPM cells that were treated with CFMs, in particular CFM-4 or CFM-5, however, revealed a robust increase in podoplanin peptides of 75 kDa and higher sizes (Figure 8B, C). Because of the extensive post-translational modifications such as glycosylation, the podoplanin is often expressed as peptides of 50 kDa or higher molecular weights whereas its cDNA encodes for a peptide of 162 aminoacids with an expected molecular mass of ∼18–20 kDa. To confirm whether CFM-4 treatments induced expression of the 75 kDa and higher sized podoplanin peptides, we conducted additional western blot analysis of lysates from the CFM-1 or CFM-4-treated MPM cells utilizing a second anti-podoplanin rat monoclonal antibody NZ-1.2. Although CFM-4-treatment caused a modest increase in levels of the 50 kDa podoplanin peptide in the lysates analyzed with NZ-1.2 antibody, both the NZ-1.2 and D2-40 monoclonal antibodies showed a robust increase in a 75 kDa peptide in the CFM-4-treated cells (supplementary figure 1). These data strongly suggest that CFM-4 stimulates expression of a 75 kDa-sized podoplanin peptide in MPM cells. Given that podoplanin expression is often associated with cancer cell motility, migration and invasion, a recent report demonstrated that PKA phosphorylation of serines in the intracellular tail of podoplanin infact interfered with the ability of podoplanin to regulate cell motility and migration [52]. Since CFM-4 activated p38 and JNK SAPKs (figure 4), and SAPKs are proline-directed serine/threonine kinases that phosphorylate serine or threonines that are followed by proline amino acid in their substrates, and the fact that podoplanin proteins of human, mouse and rat origins all have a conserved C-terminal serine that is followed by proline, it is likely that stress signaling induced by CFM-4 not only enhances podoplanin expression but also its serine phosphorylation to attenuate podoplanin-dependent motility and invasion signaling. Western blot analysis of immunoprecipitated, serine phosphorylated proteins revealed robust serine phosphorylation of podoplanin peptides in CFM-4-treated MPM cells while a modest podoplanin phosphorylation was also noted in the CFM-1-treated cells (figure 8D) when compared with their untreated counterparts. Given that there is a conserved lysine residue within the short cytoplasmic domain of podoplanin proteins of human, rat, and murine origins (VVMxKxSGRxSP), we next clarified whether the cytoplasmic region of podoplanin was also ubiquitinated following its serine phosphorylation in the presence of CFM-4. Many cellular proteins are well known to be ubiquitinated on the lysine residues following phosphorylation of the neighboring serine and/or threonine residues. Lysine48-linked ubiquitination of proteins often serves as a signal for their proteasomal degradation while lysine63-linked ubiquitination is associated with non-proteolytic functions such as signal transduction [13], [ 53], [ and 54]. Western blot analysis of immunoprecipitated, lysine63-linked proteins revealed presence of a ∼75 kDa podoplanin in lysates derived from MPM cells that were treated with CFM-1 or CFM-4 (figure 8D). Similar western blot analysis of immunoprecipitated, lysine48-linked proteins failed to indicate presence of podoplanin (not shown). Together, these data suggest that lysine63-linked ubiquitination is involved in increased podoplanin expression following CFM-4 treatments, while the phosphorylated podoplanin, in turn, is inhibited to signal for cellular motility and invasion processes. In addition, western immunoblot analysis of cisplatin or CFM-treated MPM cells showed robust cleavage of vimentin when compared with their untreated counterparts (figure 8B, C). Collectively, the data in figure 8 suggest that CFM-4 treatments likely impact cellular motility and invasion-associated functions of podoplanin and vimentin proteins in the MPM cells.

**Figure 9:**
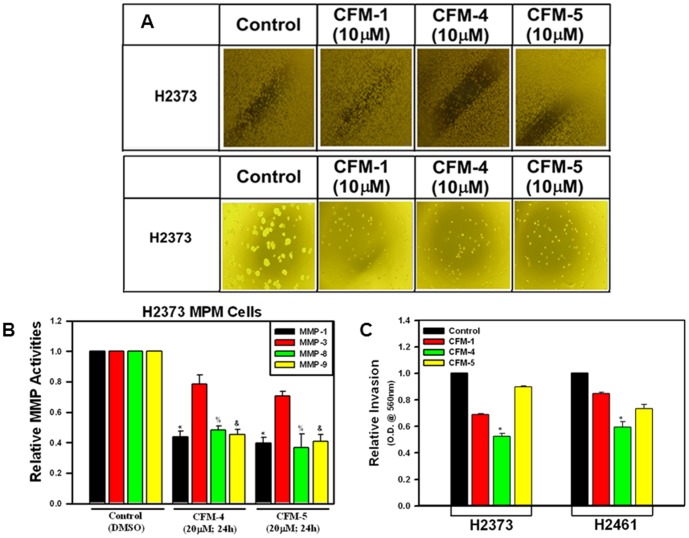
CFMs inhibit MPM Cell Growth in Soft Agar, invasion and MMP activities. (A) MPM cells were either untreated (Control) or treated with indicated dose of respective CFMs, and were subjected to the subjected to the scratch assays (indicated as wound healing assay; upper panel) or soft-agar assay (lower panel). The cells in the scratch assay or the colonies of cells in soft-agar were photographed as described in Methods. Representative photomicrographs of untreated and CFM-treated H2373 cells are shown. (B) The H2373 cells were either untreated [Control (DMSO)] or treated with CFM-4 or CFM-5 for noted dose and time. Cell lysates were analyzed for activities of various MMPs as detailed in Methods. The data in the histogram represents means of the activities of the noted MMPs from three independent experiments; bars, S.E. (*, %, and &, p = <0.05 relative to respective MMP activities in Control cells). (C) The MPM cells were separately seeded in chambers with matrigel-coated membranes, and treated with buffer (Control) or with 10 µM dose of respective CFMs as noted in Methods. Live cells migrating across the matrigel-coated membranes were dissociated, and quantitated by an MTT-based assay. The columns in histogram represent MTT OD of the CFM-treated MPM cells relative to untreated controls. (*, p = <0.02 relative to buffer-treated (Control) cells).

We further clarified the extent CFMs affect the biological properties of the MPM cells by performing wound-healing, soft-agar, and matrigel-invasion assays, respectively, as detailed in Methods. Since 20 µM dose of CFM-4 and -5 caused extensive apoptotic cell death (see figures 1 and 2), and the fact that the wound healing, soft-agar colony formation, and invasion assays were performed over the treatment periods longer than 24 h, we utilized a lower, 10 µM dose of each CFM to minimize interference from apoptosis in these assays. Presence of CFM-4 or -5 prevented the H2373 MPM cells from growing in the areas of wound created by a scratch, and also caused a greatly reduced number and size of their colonies in soft agar when compared with their respective, untreated controls (Figure 9A). Since cellular motility and invasive processes are often also regulated by extrinsic factors in the extra-cellular matrix, and various matrix metalloproteinases (MMPs) are often activated in invasive cancers and contribute to poor prognoses, we determined whether exposure to CFMs also diminished activities of any of the MMPs. For this purpose we conducted an antibody-based array analysis to determine activation status of various MMPs in control (untreated) versus treated H2373 MPM cells as indicated in Methods. These data revealed that exposure of MPM cells to CFM-4 or CFM-5 resulted in attenuation of MMP-1, -8, and -9 activities (Figure 9B). Whether CFM-dependent attenuation of MMP activities interfered with invasive properties of the MPM cells was determined next by testing the extent to which CFMs blocked the ability of MPM cells to invade through the matrigel-coated membranes. As expected, treatment of MPM cells with CFM-4 resulted in a significantly reduced number of cells that were able to migrate across the matrigel-coated membranes (Figure 9C). Taken together, these data show that CFMs, in particular CFM-4, have the ability to interfere with MPM cell invasion and metastasis-inducing pathways.

## Discussion

The management of MPM in the clinic remains challenging due primarily to the lack of sufficient treatment options. The front-line therapeutic strategies include multi-targeted antifolate agent, pemetrexed, in combination with cisplatin, the overall prognosis of patients with MPM still remains poor. Recent studies have focused on development of small molecule or antibody-based anti-MPM approaches [49], [51], there however remains a pressing need for development and testing of new anti-MPM modalities. The studies presented here highlight anti-MPM potential of a novel class of compounds termed CFMs that we have recently identified and characterized. CFMs, in particular CFM-4, are cytotoxic toward a wide variety of cancer cells as well as their drug-resistant counterparts but do not inhibit non-tumorigenic mammary epithelial MCF-10A cells [15]. Consistent with these observations, our current studies demonstrate that CFM-4 suppressed MPM cell growth by activating apoptosis signaling as well as by diminishing the levels of key cell cycle regulatory proteins such as cyclin B1 and c-myc. In addition to stimulating CARP-1 expression and activation of pro-apoptotic SAPKs (p38 and JNK), we report for the first time that CFM-4 caused elevated levels of phosphorylated podoplanin, while down-regulating key transducers of invasion and metastasis pathways.

Post-translational modifications such as phosphorylation, acetylation, ubiquitination, and glycosylation of the proteins that are involved in transduction of cellular growth and survival signaling often play critical roles in processes of cell growth, survival and homeostasis. Although kinases such as SAPKs are activated by phosphorylation of specific amino-acids in their catalytic domain, the phosphorylation of their substrates often serves to amplify or attenuate signaling in a context-dependent manner. Expression of a number of signaling proteins is often regulated also by their ubiquitination that follows their phosphorylation. In general, the proteins that are ubiquitinated at lysine (K)63 positions are stabilized for signaling while those that undergo ubiquitination at the K48 position are degraded by the proteasome system [13], [53]. Although, CFM-4 stimulated serine phosphorylation and lysine63-linked ubiquitination of podoplanin, and the fact that both the anti-podoplanin antibodies are expected to recognize a 40–43 kDa O-linked sialoglycoprotein, and since each ubiquitin subunit is ∼8–9 kDa size, it likely that conjugation of three or more ubiquitin subunits resulted in a 75 kDa and higher molecular weight podoplanin proteins in the CFM-4-treated MPM cells. Moreover, since CFM-4 prevents CARP-1 binding with APC-2 and inhibits function of APC/C E3 ubiquitin ligase [15], and APC/C ligase is well known to inversely regulate activity of SCF ubiquitin ligase [13], [14], it is possible that SCF ligase or another similar E3 ligase that is a target of APC/C, ubiquitinates serine-phosphorylated podoplanin. If so, the phosphorylation of podoplanin will likely serve a dual purpose by interfering with its signaling for cellular motility while ubiquitination-dependent stabilization would further amplify MPM motility inhibitory properties of phosphorylated podoplanin in the presence of CFM-4.

Ability of CFMs, particularly CFM-4, to inhibit MPM cell migration and invasion is consistent with their recently demonstrated effects on the motility and migration of the medulloblastoma (MB) cells [20]. Signaling by extracellular matrix MMPs, cell surface sialoglycoprotein podoplanin, intracellular small GTP-binding p21Rac1, and vimentin proteins are well known to regulate processes of cellular motility, migration and invasion of cancer cells. Moreover, deregulated expression and/or activation of MMPs, podoplanin, p21Rac1, and/or vimentin are often associated with poor prognosis in many cancers [41]–[49]. The fact that CFM-4 exposure diminished activities of a number of MMPs, caused vimentin cleavage and suppressed p21Rac1 levels while phosphorylating and likely disabling podoplanin-dependent motility signaling in MPM cells would underscore invasion and motility inhibitory properties of this class of compounds. On the basis of our earlier findings indicating robust apoptosis induction in cancer cells by CFM-4 [15], and the fact that Cisplatin treatments failed to alter expression of p21Rac1 or podoplanin in MPM cells (figures 5, 8) coupled with our data demonstrating inhibition of a diverse metastasis signaling by CFM-4 would argue for its pleiotropic anti-cancer properties, and collectively indicate for potential of CFM-4 and/or its futuristic analogs as suitable anti-MPM agents.

Our current and recent observations [20] revealed that CFM-4 activated p38 and JNK SAPKs as well as signaling by NF-κB. A large body of literature has thus far established that context and signal-dependent activation of p38 and/or JNKs could result in cell survival or apoptosis outcomes [55]. NF-κB however has been widely recognized as a highly versatile signaling transcription factor involved in regulation of cell growth, survival, and metastasis processes. Recent studies have further highlighted a crucial role for NF-κB in determining the outcome of the DNA damage response of the cells [56], [57]. NF-κB signaling was found to promote cell survival in the presence of a low to moderate levels of DNA damage while extensive DNA damage provoked TNFα-mediated JNK3-dependent apoptosis. A number of recent publications have further suggested possibility of NF-κB signaling in promoting apoptosis [23]–[25]. The nuclear to cytoplasmic signaling by NF-κB essential modulator (NEMO/IKKγ) plays a critical role in activation of canonical NF-κB pathway following DNA damage. Since CFM-4 caused diminished levels of ABIN1 (A20 binding inhibitor of NF-κB; aka TNIP1) in both the MPM (figure 6) and MB cells [20], it conceivable that loss of ABIN1 facilitates formation and/or activation of the IKK. The active IKK in turn targets IκBs to promote NF-κB nuclear translocation and transcriptional activation of its target genes. Together with the facts that CFM-4 exposure of the MPM (figure 6) and MB cells [20] also resulted in loss of IκBs would suggest that the canonical NF-κB pathway was activated by this agent. Whether activation of p38, JNK1/2, and/or NF-κB by CFM-4 play a role in apoptosis of MPM cells or function as defensive mechanisms to ensure MPM cell survival and recovery from CFM-4 treatments, and to the extent CFM-4 exposure also caused damage to the cellular DNA remain to be clarified. Nevertheless, CFM-4 was more effective in inhibiting growth of MPM cells when compared with Cisplatin (figure 1), and although both the agents suppressed MPM growth by promoting apoptosis, it is likely that activation of JNK1/2 as well as NF-κB pathways, coupled with loss of cyclin B1, c-myc, and p21Rac1, and phosphorylation of podoplanin contribute to the superior anti-MPM effects of CFM-4.

In summary, the data presented here support our working hypothesis that CFMs activate multiple cell growth inhibitory and apoptosis pathways to suppress MPM cell growth, survival and metastasis processes, and underscore their potential as novel class of anti-MPM agents.

## Supporting Information

Figure S1 **Podoplanin expression in H2373 MPM cells.** MPM cells were either untreated (Control), treated with CFM-1 or CFM-4 for noted dose and time. Two sets of cell lysates (50 µg protein/lane) were electrophoresed on 10% SDS-PAGE gel and proteins transferred to nitrocellulose membrane as in methods. The membrane containing one set of protein lysates was probed with anti-podoplanin antibody NZ-1.2 (autoradiogram on the left) and the membrane containing the second set of lysates was probed with anti-podoplanin D2-40 antibody (autoradiogram on the right). Both the membranes were then probed with anti-actin antibody to assess loading.
(TIF)
